# Prevalence of chronic kidney disease in diabetic adult out-patients in Tanzania

**DOI:** 10.1186/1471-2369-14-183

**Published:** 2013-08-31

**Authors:** Mubarakali N Janmohamed, Samuel E Kalluvya, Andreas Mueller, Rodrick Kabangila, Luke R Smart, Jennifer A Downs, Robert N Peck

**Affiliations:** 1 Department of Internal Medicine, Bugando Medical Centre, Mwanza, Tanzania; 2 Department of Tropical Medicine, Medical Mission Hospital, Wuerzburg, Germany; 3 Center for Global Health, Weill Cornell Medical College, New York, NY, USA

**Keywords:** Diabetes mellitus, Microalbuminuria, Proteinuria, CKD, Chronic kidney disease, Sub-Saharan Africa

## Abstract

**Background:**

The number of adults with diabetes mellitus is increasing worldwide, particularly in Asia and Africa. In sub-Saharan Africa, renal complications of diabetes may go unrecognized due to limited diagnostic resources. The prevalence of chronic kidney disease (CKD) among adult diabetics in sub-Saharan Africa has not been well described.

**Methods:**

This study was conducted at the diabetes mellitus clinic of Bugando Medical Centre in Mwanza, Tanzania. A total 369 consecutive adult diabetic patients were enrolled and interviewed. Each patient provided a urine sample for microalbuminuria and proteinuria and a blood sample for serum creatinine level. Estimated glomerular filtration rate (eGFR) was calculated using the Cockroft-Gault equation. CKD was staged according to the Kidney Disease Improving Global Outcomes system.

**Results:**

A total of 309 (83.7%) study participants had CKD; 295 (80.0%) had significant albuminuria and 91 (24.7%) had eGFR < 60 ml/min. None of these patients were aware of their renal disease, and only 5 (1.3%) had a diagnosis of diabetic nephropathy recorded in their file. Older age was significantly associated with CKD in this population [OR 1.03, p = 0.03, 95%CI (1.00-1.05)].

**Conclusions:**

Chronic kidney disease is highly prevalent among adult diabetic outpatients attending our clinic in Tanzania, but is usually undiagnosed. Nearly ¼ of patients had an eGFR low enough to require dose adjustment of diabetic medications. More diagnostic resources are needed for CKD screening among adults in Tanzania in order to slow progression and prevent complications.

## Background

Diabetes mellitus is a steadily growing global epidemic. The World Health Organization (WHO) predicted that the number of people living with this disease would reach 221 million by 2010 and will further increase to 300 million by 2025 with the majority of new cases occurring in Asia and Africa [1,2]. In sub-Saharan Africa alone, the number of people with diabetes is projected to increase from 7 million in 2000 to 18 million in 2030, a regional increase of 161% [3]. Additionally, complications of diabetes mellitus are more prevalent among patients with diabetes in Africa as compared to the developed world due to late presentation, limited screening and diagnostic resources, poor glycemic control, and inadequate treatment of complications at an early stage [4,5].

Chronic kidney disease (CKD) is one of the most common complications of diabetes mellitus. Screening for CKD is not routinely performed in many diabetic clinics in sub-Saharan Africa due to limited diagnostic resources. In particular, microalbumin testing is available in very few centers [4].

Few studies have described the burden of CKD among adults with diabetes in sub-Saharan Africa. Therefore, in this study we aimed to determine the prevalence, awareness and factors associated with CKD among diabetic adult outpatients attending our clinic in Tanzania for routine care using both serum creatinine and urine microalbumin testing.

## Methods

### Study setting

This was a cross-sectional study conducted at the outpatient diabetes clinic of Bugando Medical Centre (BMC) in Mwanza, Tanzania. BMC is the zonal hospital for the Lake Victoria region in northwest Tanzania, and it serves a population of approximately 13 million people. Our outpatient diabetes clinic follows approximately 1000 children and adults from the Mwanza region for primary diabetes care. In Tanzania, the large majority of diabetic patient care is provided at referral and regional hospital clinics as these are the only facilities that have a reliable supply of the necessary equipment and drugs for diagnosis and management of diabetes mellitus. Referral hospitals like BMC provide primary diabetes patient care in the Lake Zone region.

### Study population

We consecutively enrolled patients between October 2011 and March 2012. All adults (≥18 years) with known diabetes mellitus who attended the BMC clinic during this time period were eligible for this study. Those who gave informed consent and agreed to provide samples of urine and blood were enrolled. In order to determine the prevalence of CKD within a confidence interval of +/- 5%, a required sample size of 368 was calculated using the Kish-Leslie formula.

### Study design and data collection

At the time of enrollment, all study participants were asked to complete a pre-validated, standardized questionnaire that included sociodemographic and clinical characteristics related to diabetes mellitus. The sociodemographic and clinical information collected in the questionnaire were as follows: age, gender, occupation, level of education, body mass index (BMI), duration of diabetes, hypoglycemic agent/s used, presence and treatment of hypertension, smoking/alcohol intake, HIV serostatus, family history of kidney disease, and urinary symptoms. In order to ascertain awareness of renal disease within the study population at the time of enrollment, we asked the following question, “Have you been previously informed about a decrease in your kidney function or about a kidney problem by your doctor or nurse?”

Chart review was used to determine whether patients had prior diagnoses of hypertension or renal disease. Patients were labeled hypertensive if their charts documented a diagnosis and/or treatment of hypertension. For those attending the clinic for the first time on the day of study enrollment, the diagnosis of hypertension was made if systolic blood pressure was >140 mmHg and/or diastolic blood pressure was >90 mmHg.

Random blood glucose measurement was performed (Ascensia Glucometer, Bayer Healthcare, Germany). A fresh urine sample was tested for microalbumin using a dipstick (Micral B, Roche, Germany). Patients were considered to have microalbuminuria if the urine albumin concentration was above 20 mg/L, as per instructions provided by the manufacturer and according to prior research [6,7]. A second urine dipstick was used to test for overt proteinuria (Multistix 10SG, Siemens, USA). Serum creatinine was also measured using a Cobas 400 clinical chemistry machine (Roche, Germany), calibrated by the creatinine Jaffe 2 method. An estimated glomerular filtration rate (eGFR) was calculated using the Cockroft-Gault equation in order to remain consistent with other studies done in our region [6,8]. After calculating the eGFR, patients were classified according to the Kidney Disease Improving Global Outcomes (KDIGO) system [9]. CKD was defined as an eGFR ≤60 ml/min/1.73 m^2^ or evidence of kidney damage (microalbuminuria or overt proteinuria).

### Data analysis

The primary outcomes were prevalence and severity of CKD within the study population according to the KDIGO classification. Data was entered into Microsoft Excel and analyzed using STATA version 10 (STATA Corporation, San Antonio, Texas). Categorical variables were described as proportions (%), and continuous variables were described as medians [interquartile range]. In the univariate analysis, Fisher’s exact and chi-squared tests were used for categorical and binary variables respectively and the Wilcoxon rank-sum test was used for continuous variables. Predictors with a p < 0.10 were included in the multivariate model using logistic regression.

### Ethical issues

Ethical clearance for the study was obtained both from the Research and Publications Committee BMC and the Institutional Review Board of Weill Cornell Medical College.

## Results

### Enrollment

Between October 2011 and March 2012, 960 diabetic patients attended the BMC clinic. Out of these, 591/960 were ineligible because they were <18 years old. All of the remaining 369 adult diabetic patients consented, were enrolled, and provided urine and blood samples.

### Study cohort

Of the 369 study participants, 197 (53.4%) were female, and 258/369 (70.3%) were from Mwanza City. The median age was 54 years [45-62 years]. The median BMI was 25.6 kg/m^2^ [22.6 – 29.6 kg/m^2^], the median duration of diabetes was 6 years [3 – 11 years], and 347/369 (93.8%) had type 2 diabetes mellitus. Among these diabetic adults, 212/369 (57.5%) had co morbid hypertension but only 35/212 (16.5%) had achieved target blood pressure control (<130/80 mmHg). Among our diabetic patients, 199/369 (53.9%) were on an Angiotensin Converting Enzyme inhibitor (ACE-I) or an Angiotensin II Receptor Blocker (ARB). See Table 1 for baseline characteristics.

**Table 1:** Demographic and clinical characteristics of 369 patients attending the diabetes clinic at BMC

**Characteristic**		**Number (%) or Median [IQR]**
**Female Gender**		197 (53.4%)
**Residence:**	Within Mwanza City	258 (70.3%)
**Age (years)**		54 [45 – 62]
**Education**	Primary or Less	232 (62.9%)
	Secondary or More	137 (37.1%)
**Occupation:**	Peasant	96 (26.0%)
Others (Employed, Business, Housewife, Retired)		273 (74.0%)
**Body Mass Index (kg/m**^**2**^**)**		25.6 [22.6 – 29.6]
**Duration of Diabetes (years)**		6 [3 – 11]
**Diabetes Medication:**	Oral	205 (55.6%)
	Insulin	148 (40.2%)
	Insulin + OHGA	4 (1.1%)
**Hypertension Prevalence**		212 (57.5%)
**Duration of Hypertension (years)**		1 [0 – 7]
**Anti-Hypertensive Medication:**	None	158 (42.9%)
	ACE-I or ARB	128 (34.8%)
	Other	11(3.0%)
	ACE-I or ARB + Other	71 (19.3%)
**Current Smoking**		12 (3.3%)
**Current Alcohol Consumption**		38 (10.3%)
**Quantity of Alcohol Among Drinkers (units/wk)**		3 [1 – 6]
Previously Diagnosed Renal Disease*		5 (1.3%)
**Systolic Blood Pressure (mmHg)**		130 [120 – 150]
**Diastolic Blood Pressure (mmHg)**		80 [70 – 90]
**Random Blood Glucose (mmol/L)**		9.8 [6.7 – 14.1]

*ACE-I* angiotensin converting enzyme inhibitor.

*OHGA* Oral Hypoglycemic Agents.

* Pre-existing diabetic nephropathy as depicted by prior documentation of proteinuria, abnormally elevated serum creatinine levels and enlarged, echogenic kidneys on renal sonography.

### Prevalence of CKD

Of all study participants, 309/369 (83.7%) had CKD and 91/367 (24.7%) had eGFR < 60 mL/min. A total of 295/369 (80.0%) had moderately to severely increased albuminuria (Table 2); 126/369 (34.1%) had overt proteinuria and 169/369 (45.8%) had microalbuminuria alone. Prior to enrollment, none of the study participants were aware of the presence of their CKD and only 5 (1.3%) had a diagnosis of CKD recorded in their charts.

**Table 2:** Primary renal outcomes of 369 patients attending the diabetes clinic at BMC

**Outcome**	**Number (%) OR Median [IQR]**
**Total with Abnormal Albuminuria**	295 (79.9%)
Overt Proteinuria	126 (34.1%)
Microalbuminuria Only	169 (45.8%)
**Creatinine Clearance (ml/min/1.73 m**^**2**^)*	87.4 [60.9 – 116.6]
**KDIGO GFR Category**	
CrCL > 90 (G1)	169 (46.0%)
CrCL 60 – 89 (G2)	107 (29.2%)
CrCL 45 – 59 (G3a)	44 (12.0%)
CrCL 30 – 44 (G3b)	33 (8.9.0
CrCL 15 – 29 (G4)	12 (3.3%)
CrCL < 15 (G5)	2 (0.5%)
CKD by KDIGO Definition*	309 (84.2%)

Note: 5 patients (1.3%) had previously diagnosed renal disease.

*Nonmissing data were included in each calculation.

### Factors associated with CKD

By univariate analysis, the following variables were found to be associated with chronic kidney disease: older age (p = 0.001), gender (p = 0.09), use of only oral hypoglycemic agents (p = 0.03), presence of hypertension (p = 0.07), and duration of hypertension (p = 0.03). The above variables were then subjected to multivariate analysis and only one variable, older age, was found to be significantly associated with CKD (OR 1.03 [95% CI = 1.00-1.05], p = 0.03).

## Discussion

CKD was almost ubiquitous among the Tanzanian adults with diabetes mellitus attending our clinic. More than 80% of patients had CKD and nearly 25% had an eGFR <60 ml/min/1.73 m^2^. This highlights the importance of implementing routine screening for CKD among adult diabetics in sub-Saharan Africa [10], a region where screening for nephropathy among adult diabetics is often not performed at all due to limited resources [4,7,10]. Early screening detection would allow, more aggressive measures to be taken (stricter glycemic control and use of Angiotensin II receptor blockade) to reverse microalbuminuria and/or prevent further adverse renal and cardiovascular outcomes [11,12].These findings are particularly concerning in a resource-limited region where the treatment options for end stage renal disease are severely limited. From our hospital, for example, the nearest hemodialysis unit is >500 km away.

The prevalence of moderately to severely increased albuminuria was particularly high in our study population. The rate of overt proteinuria in this study (34.1%) was even higher than other studies in the sub-Saharan Africa, with the highest rate in comparative studies being 20% [10]. The prevalence of microalbuminuria (45.8%) was comparable to other studies from sub-Saharan Africa [5,7,10,13-15], but higher than studies from the United States, Europe and Asia where the prevalence of microalbuminuria has ranged from 10-32% and the prevalence of overt proteinuria has ranged from 2.4-29% [16-23].

In addition to a high prevalence of albuminuria, nearly 25% of our adult Tanzanian diabetic patients had an eGFR <60 ml/min/1.73 m^2^[22]. An eGFR <60 ml/min/1.73 m^2^ represents loss of half or more of adult kidney function and is an ominous finding in a setting where few options exist for advanced kidney disease. Such a low eGFR also has implications for drug therapy for diabetes mellitus and, if undetected, could lead to complications such as lactic acidosis from metformin or hypoglycemia from sulfonylureas or insulin. In our clinical experience, drug toxicities due to lack of adjustment of drug therapy in the setting of renal dysfunction are a common cause of admission among Tanzanians with diabetes.

Older age was significantly associated with CKD in our study population. This is consistent with other studies [24]. However, other studies have also found other factors to be associated with CKD among diabetic adult use of insulin [13,17,24]. In our study, the presence and duration of hypertension were significantly associated with CKD by univariate analysis. These factors were not significant by multivariable analysis. This was likely due to the very small number of patients without CKD in our study (n = 60), which produced low statistical power to detect associated factors.

The high prevalence of advanced nephropathy among our Tanzanian adult diabetic patient may be related to several factors. First, patients in our setting may be diagnosed with diabetes at a later stage of their disease [4]. The median length of diabetes reported in the study was 6 years, but, according to our clinical experience, these patients likely had diabetes mellitus for many years before diagnosis but only presented at a later stage due to limited access to health care, cost and a higher tolerance for diabetic symptoms. It is known from the United Kingdom Prospective Diabetes Study (UKPDS) that type 2 diabetics progress to microalbuminuria at a fairly predictable rate (about 25% after 10 years of disease). This progression continues and will eventually lead to macroalbuminuria/overt proteinuria and finally a decrease in eGFR [25]. Of note, non-diabetic renal disease (NDRD) may also coexist in this patient population [26,27], and due to limited resources, could not be excluded.

One weakness of this study is the possibility of referral bias since this study was conducted at a referral hospital. On the other hand, most diabetes mellitus care in Tanzania occurs at referral hospitals and almost all of the patients in our study were receiving primary care (and not referral) services at our hospital. Another limitation of this study was the use of the Cockroft-Gault equation to calculate eGFR. Other equations, such as the MDRD equation, may be superior in estimating the GFR but the best eGFR equation for East African adults has not yet been determined. Lastly, as mentioned above, the presence of NDRDs could not be excluded.

## Conclusions

In conclusion, our study identified an alarmingly high prevalence of CKD among the Tanzanian adult diabetics attending our clinic. More than 80% of our diabetic outpatients had CKD and nearly 25% had an eGFR <60 mL/min/1.73 m^2^. These results highlight the urgent need for regular nephropathy screening among African diabetic adults in order to prevent progression to chronic renal disease [10]. This is particularly important in sub-Saharan Africa where few treatment options exist for end stage renal disease, and it will become even more important as the incidence of diabetes continues to increase in this region.

## Abbreviations

ACE-I: Angiotensin converting enzyme inhibitor; ARB: Angiotensin II receptor blocker; BMC: Bugando Medical Centre; BMI: Body mass index; CKD: Chronic kidney disease; eGFR: Estimated glomerular filtration rate; KDIGO: Kidney disease improving global outcomes; NDRD: Non-diabetic renal disease; OHGA: Oral hypoglycemic agent; OR: Odds ratio; UKPDS: United Kingdom prospective diabetes study; WHO: World Health Organization.

## Competing interests

The authors declare that they have no competing interests.

## Authors’ contributions

MNJ conceived of the idea, designed the study, collected and analyzed the data, and drafted the manuscript. SEK participated in the design of the study. AM assisted with the design of the study. RK assisted with the design of the study. LRS edited the manuscript. JAD assisted with data analysis and edited the manuscript. RNP assisted in design and coordination of the study, data analysis, and editing of the manuscript. All authors read and approved the final manuscript.

## Pre-publication history

The pre-publication history for this paper can be accessed here:

http://www.biomedcentral.com/1471-2369/14/183/prepub
